# Post-operative Therapeutic Management in an Adult Female With Cystic Hygroma: A Rare Case

**DOI:** 10.7759/cureus.50604

**Published:** 2023-12-15

**Authors:** Dhanashree Upganlawar, Prasad P Dhage, Priyanka A Telang, Neha R Badwaik

**Affiliations:** 1 Community Health Physiotherapy, Ravi Nair Physiotherapy College, Datta Meghe Institute of Higher Education and Research, Wardha, IND

**Keywords:** cryotherapy, post-op rehabilitation, cervical physiotherapy, physical therapy, cystic hygroma

## Abstract

Birth abnormalities affecting the lymphatic system include cystic lymphangiomas. They are rare in adults and typically happen in childhood. The cause of adult cystic hygroma (CH), which has a benign nature, is yet unknown. Seventy-five percent of lymphatic malformations have a CH as their primary site of origin in the head and neck area. We describe a 36-year-old female case of cervical cystic lymphangioma who complained of swelling on the left side of her neck for two years. There was no prior history of fever, trauma, weight loss, appetite loss, discharge, or swallowing difficulties. The doctor advised investigations like computed tomography neck, ultrasound sonography neck, etc., and was diagnosed with cystic lymphangioma. Early physiotherapy seems beneficial in preserving shoulder movement and minimizing pain in individuals. Cryotherapy is useful in treating patients with lymphangioma after surgery to reduce pain and swelling. This clinical case study demonstrates how patients with cystic lymphangiomas can benefit from physical treatment and regain their functional independence.

## Introduction

Cystic lymphangioma is also referred to as cystic hygroma (CH). CH are benign, non-cancerous tumors arising from the lymphatic system, which develop in the neck. They can also develop following trauma or infection [1]. They can manifest themselves in a variety of locations, including the spermatic cord level, the mediastinum, the breast, the cervical area, the abdomen, the inguinal region, and the spleen [2]. Approximately one out of every 2000 to 4000 live births have lymphangiomas [3]. The majority of CH cases are seen in children under the age of two. They are extremely uncommon in adults and are likely to be caused by the growth of lymphatic capillaries in reaction to head and neck infections or trauma [4]. The lesion is typically unilateral and doughy to the touch [5]. The posterior region is where neck CHs are most frequently discovered, whereas inflammatory, metastatic adenopathies or lymphoproliferative disorders are the most typical pathologies [6].

In areas where it compresses nearby tissue, symptoms may appear. There could be obstructive symptoms including airway blockage, dysphonia, and dysphagia [7]. Cystic lymphangiomas can be classified as congenital or acquired. Lymphatic channels are improperly connected to the main drainage ducts, resulting in congenital lymphangiomas. When previously normal lymphatic pathways are disrupted as a result of surgery, trauma, cancer, or radiation therapy, acquired lymphangiomas develop [8]. The diagnosis of adults is thought to be more difficult than that of children, and the ultimate diagnosis is typically dependent on post-operative histology [8]. Although there are numerous treatment options for lymphangiomas, surgical excision is the most popular one. Other options include radiotherapy, sclerotherapy, cryotherapy, electrical stimulation, steroid therapy, and the use of laser therapy [9]. There is evidence to report the effect of physiotherapy treatment protocols on CH survivors during their cancer treatment [10]. Physical therapy, which uses manual techniques, exercise regimens, and electrotherapeutic modalities, is used to treat joints which are having movement restrictions [11].

## Case presentation

**Figure 1:**
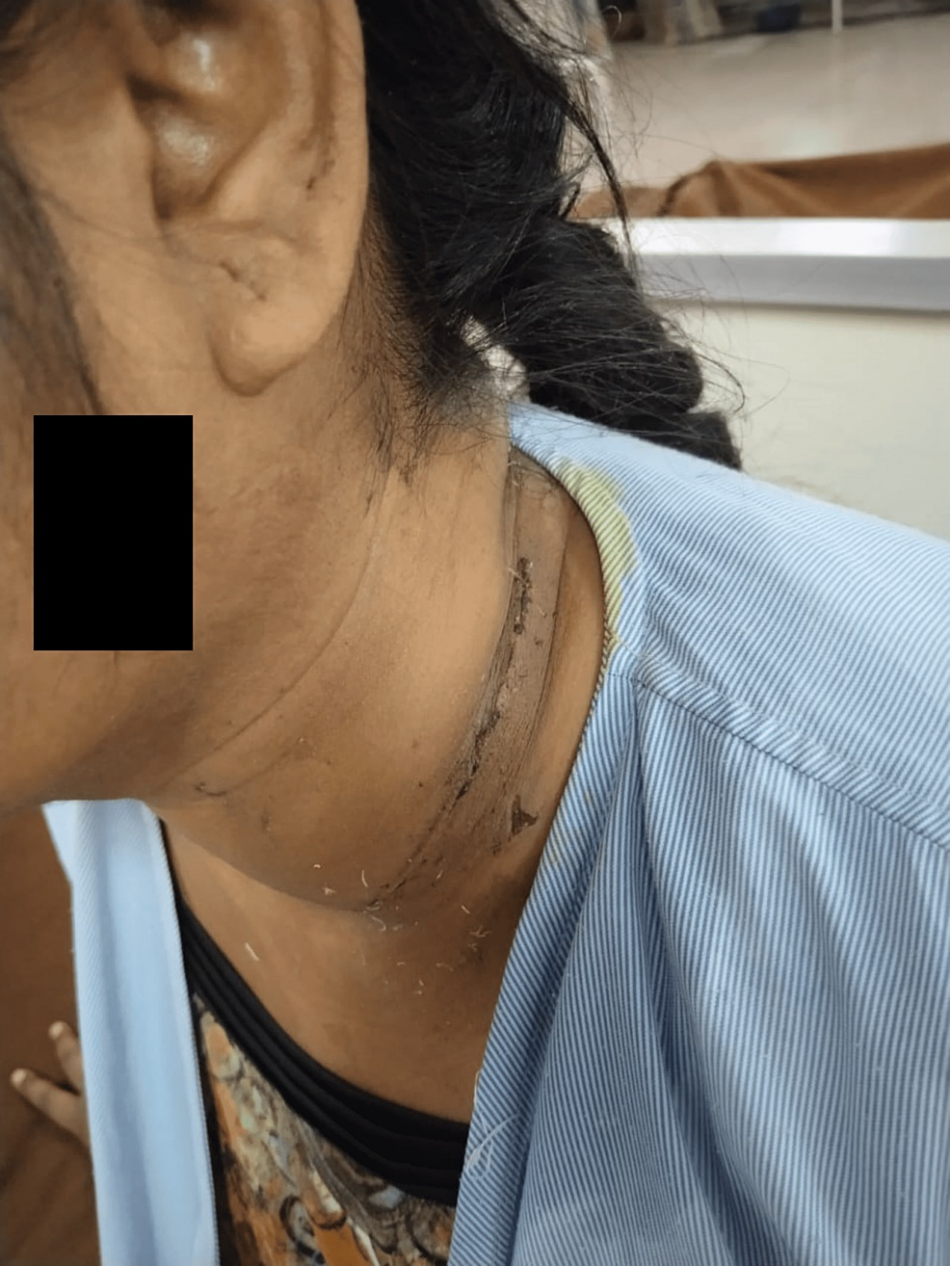
Sutures can be seen on the anterior surface of the neck

A 36-year-old female reported to the hospital with the primary complaint that her neck was swollen on the left side for two years. When the patient first observed a swelling over the left side of her neck two years ago, it had an insidious onset and was gradually worsening in character. There was no previous history of temperature, trauma, loss of weight, decreased appetite, discharge, or swallowing difficulties. The patient was a known case of tuberculosis six years back which was confirmed through the Mantoux test and was managed for the same. On inspection, sutures were seen (Figure 1), and on palpation, firm swelling was present and tenderness was grade 2 according to the tenderness grading scale that is patient winces due to pain (the grades of tenderness are as follows grade 0 is no tenderness, grade 1 is patient complains of pain, grade 2 is patient complains of pain and winces, grade 3 is patient winces and withdraws the hand and grade 4 is the patient does not allow to touch the affected part). On local examination, 8 X 6 cm swelling was present on the anterior surface of the neck in the middle and lower 1/3 towards the left of the midline. There were no dilated veins, discharge, or sinuses. There was a tightness of the trapezius muscle in this patient.

Clinical findings

On examination, the range of motion (ROM) of the cervical and shoulder joint was taken before the physiotherapy treatment was started and it showed a reduction in the joint ROM of the involved or the operated side. Table 1 represents the ROM of the joints. Table 2 shows the manual muscle testing (MMT) of the musculature which was significantly reduced for the muscles surrounding the cervical region.

**Table 1:** Range of motion

Joints/movements (in degree)	Right	Left
Cervical flexion	0^o^-50^o^	
Extension	0^o^-60^o^	
Lateral flexion	0^o^-20^o^	0^o^-25^o^
Rotation	0^o^-60^o^	0^o^-55^o^
Shoulder flexion	0^o^-180^o^	0^o^-110^o^
Abduction	0^o^-180^o^	0^o^-130^o^

**Table 2:** Manual muscle testing 0: no contraction; 1: flickering of contraction; 2: full ROM, gravity eliminated; 3: full ROM against gravity; 4: full ROM against gravity with minimum resistance; 5: full ROM against gravity with maximum resistance ROM: Range of motion

Muscles	Right	Left
Cervical flexors	3/5	
Extensors	3/5	
Lateral flexors	3/5	3/5
Rotators	4/5	3/5
Shoulder flexors	4/5	3+/5
Extensors	4/5	3/5
Abductors	4/5	3/5

Timeline of events

On November 10, 2022, the patient was admitted to the surgery ward with the complaint of a swollen neck on the left side. On November 11, 2022, ultrasonography (USG) of the neck was carried out and showed well-defined hypoechoic lesions. On November 18, 2022, other investigations were carried out and the doctor suggested that the patient was fit for the surgery. On November 19, 2022, the patient was taken for surgery where the excision of the cyst was done. After four days of surgery on November 23, 2022, the physiotherapy call was noted and the physiotherapy session was carried out. After the successful recovery of the patient on December 5, 2022 patient was discharged.

Investigations

**Figure 2:**
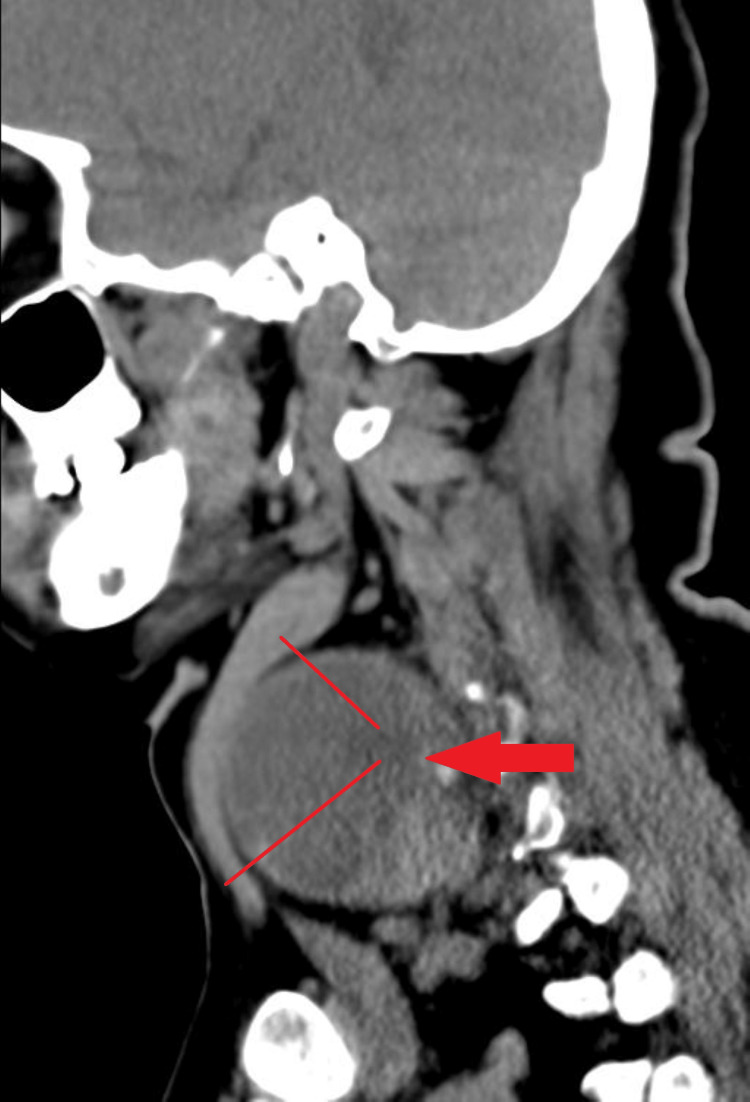
Computed tomography scan image of this patient Red lines and an arrow represent the tumor.

A computed tomography scan of the neck was performed which showed a single well-circumscribe peripherally enhancing multi-loculated cystic lesion in the left anterior cervical region surrounded by cervical vertebrae and muscle-like sternocleidomastoid muscle possibility of cystic lymphangioma that is shown in Figure 2. USG neck was also performed which showed a well-defined hypoechoic solid adjacent to the carotid space with multiple septations and internal debris, probably benign. After the investigations, the patient was diagnosed with cystic lymphangioma/CH on the left side of the neck. Before performing the histological examination the differential diagnoses were salivary gland swelling, thyroglossal cyst, and lipoma. The examinations showed large, uneven, dilated lymphatic channels with collagenous stroma separating them were seen in the histopathology. The channels were lined by a single layer of benign flattened endothelium. The stroma revealed a strong infiltration of lymphocytic cells in addition to the development of lymphoid follicles. CH was the confirmed diagnosis.

Physiotherapy management

**Figure 3:**
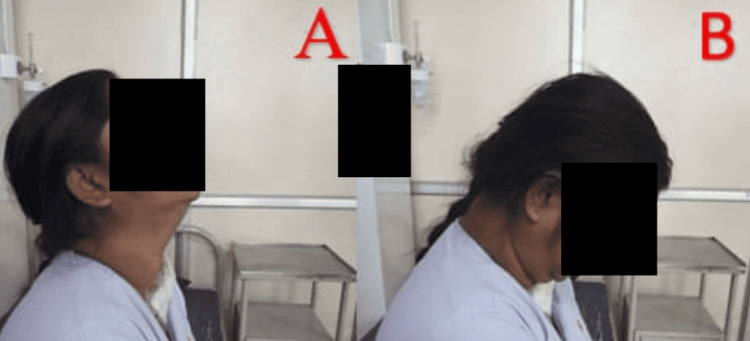
Patient performing an active cervical range of motion Cervical extension (A) and cervical flexion (B)

**Figure 4:**
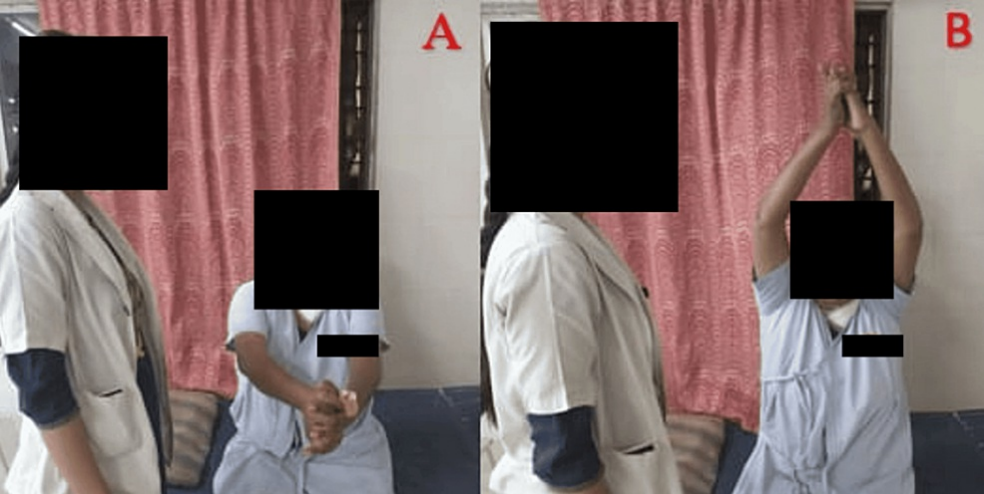
Patient performing thoracic expansion exercise Initiation of thoracic expansion (A) and completion of thoracic expansion (B)

As part of post-operative physiotherapy, the patient is treated for pain, and contractures, improves ROM, prevents chest complications, and is made independent to conduct daily life tasks, and improves her quality of life. The rehabilitation regimen is given in Table 3. Figure 3A, 3B, 4A, 4B shows the patient being rehabilitated.

**Table 3:** Physiotherapy rehabilitation protocol AROM: Active range of motion; cc: Cubic centimeter

Goals	Rehabilitation protocol	Regimen
Patient education	Encourage the patient to exercise their upper and lower limbs after surgery to learn more about the condition and how to manage it post-operatively.	Pre- and post-operatively follow up once a week
To reduce pain	Cryotherapy (at -110^o^C for four minutes) around the suture site wrapped with clothes.	Three times a day
To avoid muscle tightness and contracture	Passive stretching of the trapezius, cervical AROM exercises include lateral rotation and cervical side rotation, shoulder shrugs, and scapular sets.	Three repetitions with 15 seconds hold three times a day
To avoid chest complication	Exercises such as spirometry, deep breathing exercises, pursed lip breathing, and thoracic expansion should be done.	This was done pre- and post-operatively. Spirometry at 600-900-1200 cc. Thoracic expansion and breathing exercise 10 repetitions X 2 sets three times a day

Follow-up and outcome measures

The effectiveness of the rehabilitation was evaluated using ROM (Table 4), and MMT (Table 5). We can see the improvement in this patient by following regular exercise protocol. The ROM of the cervical joint was comparatively increased by the end of week two as compared to that of week one. MMT was also performed after the period of two weeks of treatment protocol to assess the improvement in the strength of the muscles, which was seen to be improved and the patient was able to perform the movements independently. Neck disability index was another outcome measure taken (Table 6).

**Table 4:** Range of motion post-treatment

	Week 1		Week 2	
Joints/movements (in degree)	Right	Left	Right	Left
Cervical flexion	0^o^-65^o^		0^o^-75^o^	
Extension	0^o^-65^o^		0^o^-70^o^	
Lateral flexion	0^o^-25^o^	0^o^-35^o^	0^o^-35^o^	0^o^-45^o^
Rotation	0^o^-70^o^	0^o^-65^o^	0^o^-80^o^	0^o^-75^o^
Shoulder flexion	0^o^-180^o^	0^o^-135^o^	0^o^-180^o^	0^o^-150
Abduction	0^o^-180^o^	0^o^-150^o^	0^o^-180^o^	0^o^-165^o^

**Table 5:** Manual muscle testing post-treatment 0: no contraction; 1: flickering of contraction; 2: full ROM, gravity eliminated; 3: full ROM against gravity; 4: full ROM against gravity with minimum resistance; 5: full ROM against gravity with maximum resistance ROM: Range of motion

	Week 1		Week 2	
Muscles	Right	Left	Right	Left
Cervical flexors	4/5		4/5	
Extensors	4/5		4/5	
Lateral flexors	4/5	4/5	4/5	4/5
Rotators	4/5	4/5	4/5	4/5
Shoulder flexors	4/5	4/5	5/5	4/5
Extensors	4/5	4/5	5/5	4/5
Abductors	4/5	4/5	5/5	4/5

**Table 6:** Neck disability index The neck disability index is a questionnaire that is used to determine how bad the pain in the neck is and how this is affecting the individual's daily living activity with a total score of 50 points. 0-4 points: no disability; 5-14 points: mild disability; 15-24: moderate disability; 25-34 points: severe disability; 35-50 points: complete disability

Neck disability index	
Pre-rehabilitation	18/50
Post-rehabilitation	3/50

## Discussion

Intervention such as cryotherapy at -110^o^C for four minutes and active-assisted exercises of the neck and shoulder was given progress to active resisted and lastly progress to active range of motion (AROM), strengthening exercises of cervical and shoulder musculatures starting with isometrics with the hold of 5 seconds and progress to 10 seconds of hold. Stretching of the trapezius muscle for 30 seconds for 3 sets, and breathing exercises to avoid chest complications after surgery were used to gain recovery for the patient. At the end of two weeks of physical therapy rehabilitation, there was an improvement seen in the patient.

Karadag et al. reported the successful use of a pulsed dye laser and cryotherapy combination in the treatment of two cases of lymphangioma [12]. After using cryotherapy in our case scenario, we too achieved results. According to Baggi et al., joint distraction and oscillations from AROM exercises lead to early joint mobilization, reduced discomfort, and increased nutrition supply to the joint area [13]. Following its use in our case report, we also found the AROM exercise to be helpful.

Singhavi et al. reported the successful use of shoulder physical therapy which includes active and assisted ROM, strengthening of the scapular elevators, neuromuscular retraining of the shoulder girdle muscles, passive stretching of the trapezius helps in regaining the shoulder movements, and shoulder musculature strengthening [14]. After using the above exercises we have gained successful results in our case scenario. Shanmugasundaram and Dhanasekaran used deep breathing exercises and incentive spirometry to avoid chest complications in a head-neck cancer patient in their study [15]. We also used this exercise in our case report and found it to be useful.

## Conclusions

This case study demonstrates how physiotherapy can help patients with cystic hygroma regain their independence. The patient has had CH and has a post-operative complication as a result of its excision. Physical therapy helps in regaining the range of motion of the cervical and shoulder, reducing pain, to improving the strength of cervical and shoulder musculatures. The entirety of the treatment can be given during the rehabilitation program.
